# Surgical Ventricular Restoration for Ischemic Heart Failure: A Glance at a Real-World Population

**DOI:** 10.3390/jpm12040567

**Published:** 2022-04-02

**Authors:** Serenella Castelvecchio, Valentina Milani, Federico Ambrogi, Marianna Volpe, Lucia Ramputi, Giovanni Soletti, Lorenzo Menicanti

**Affiliations:** 1 Department of Cardiac Surgery, I.R.C.C.S. Policlinico San Donato, 20097 San Donato Milanese, Italy; lucia.ramputi@grupposandonato.it (L.R.); lorenzo.menicanti@grupposandonato.it (L.M.); 2 Laboratory of Biostatistics and Data Management, Scientific Directorate, I.R.C.C.S. Policlinico San Donato, 20097 San Donato Milanese, Italy; valentina.milani@grupposandonato.it (V.M.); federico.ambrogi@unimi.it (F.A.); 3 Department of Clinical Sciences and Community Health, University of Milan, 20122 Milan, Italy; 4 Department of Cardiac Rehabilitation, I.R.C.C.S. Policlinico San Donato, 20097 San Donato Milanese, Italy; marianna.volpe@grupposandonato.it; 5 Cardiothoracic Surgery Department, Weill Cornell Medicine, NewYork-Presbyterian Hospital, New York, NY 10065, USA; gis2011@med.cornell.edu

**Keywords:** ischemic heart failure, left ventricular remodeling, surgical treatment

## Abstract

Surgical ventricular restoration (SVR) has repeatedly been suggested as a viable therapeutic strategy for ischemic heart failure (HF) patients, although the survival benefit is still debated. We investigated a real-world population treated with SVR in a single center with high case volumes. From July 2001 to June 2017, 648 patients (111 females) underwent SVR; coronary surgery was performed in 582 patients. Data were analyzed by dividing the population into two groups: Group I (371 patients operated between July 2001 and December 2007) and Group II (277 patients operated between January 2008 and June 2017). At baseline, Group I patients were more symptomatic for angina (47.4% versus 19.4%, *p* < 0.0001) and less symptomatic for HF (NYHA class III/IV, 46.3% versus 57%, *p* = 0.0071). The end-diastolic volume (106 mL/m^2^ versus 118.3 mL/m^2^, *p* < 0.0001) and the end-systolic volume (70.5 mL/m^2^ versus 81.5 mL/m^2^, *p* < 0.0001) were lower in Group I. The presence of 3-vessel coronary artery disease (CAD) was higher in Group I (73.3% versus 59.2%, *p* < 0.0001). Thirty-day mortality (6.64%) was similar in the two groups (*p* = 0.4475). The Kaplan–Meier estimate for all-cause mortality for the entire population was 13% at 2 years, 19.2% at 4 years and 36.6% at 8 years, and the probability was not different between groups (Log-rank = 0.11). In a real-world ischemic HF population, SVR may be carried out with favorable results; in patients with worse LV remodeling and less extensive CAD, SVR showed a trend toward a better outcome.

## 1. Introduction

Heart failure (HF) remains a lethal condition with a mortality rate of up to 60% within five years of diagnosis [1,2], driven mostly by the causal role of coronary artery disease (CAD) in the development of left ventricular (LV) remodeling and dysfunction. The increasing prevalence of this poor condition is related to the longevity and the improved survival after a previous myocardial infarction (MI) [3]. Coronary artery revascularization (CABG) is the first strategy in patients with chronic HF and systolic LV dysfunction (class I, level of evidence B) [4]. Surgical ventricular restoration (SVR) at the time of CABG may be performed in selected patients treated in centers with expertise (class IIb, level of evidence B) [4]. Both recommendations stem from the results of the STICH (Surgical Treatment of Ischemic Heart Failure) trial, which is more robust for the comparison of CABG versus optimal medical therapy in patients with ischemic LV dysfunction and HF [5,6]. However, it is less evident for the additional role of SVR to CABG [7]. After more than ten years after the publication of the STICH- Hypothesis 2 results, it has been well recognized that this trial enrolled a heterogeneous population of patients with CAD and low ejection fraction (EF), with moderate symptoms of either angina and HF and relative small volumes, for which the preference between different therapeutic strategies (i.e., CABG or SVR) was not so relevant [8]. However, no effort has been made to understand which group of patients would actually benefit from this procedure in the long term, leaving uncertainties among the scientific community.

The objectives of this study were (i) to assess the clinical and imaging features, surgical treatment and prognosis of a real-world ischemic HF population referred to our center over sixteen years; and (ii) to evaluate the long-term outcome. The outcome of interest of the study was all-cause mortality, including death within 30 days. 

## 2. Materials and Methods

### 2.1. Study Design

This was a retrospective study based on the database of the I.R.C.C.S. Policlinico San Donato for patients with ischemic HF undergoing cardiac surgery. The study protocol was approved by the Local Ethics Committee, according to the Italian regulatory law for observational retrospective studies. Patient consent was waived due to the processing of data in anonymized form.

### 2.2. Study Population

From July 2001 to June 2017, 648 patients (111 females (17%), median age 66 [IC 58–72]) with previous MI and LV remodeling were referred to our center for cardiac surgery. Follow-up continued through June 2018. All patients underwent SVR (carried out by a single surgeon—LM); CABG was performed in 582 patients (89.8%) and mitral valve surgery in 200 patients (30.1%). Indications for surgery were heart failure and/or angina. 

Given changes in clinical practice over time that could have resulted in differences in the baseline profile, data were analyzed by dividing the population into two groups: Group I (patients operated between July 2001 and December 2007) and Group II (patients operated between January 2008 and June 2017). Time intervals were chosen in order to have two comparable groups by number and length of average follow-up of at least 5 years. 

### 2.3. Echocardiography

A comprehensive echocardiographic assessment was performed using a GE Vivid 7 (GE Healthcare, Waukesha, WI, USA) instrument, and all 4-chamber, 2-chamber, long-axis and short-axis views were analyzed before and after surgery at a median time of 12 months from the operation. The following parameters were collected: LV diastolic diameter (mm), LV systolic diameter (mm), end-diastolic volume (EDV) index (mL/m^2^), end-systolic volume (ESV) index (mL/m^2^), EF (%), stroke volume (SV) index (mL/m^2^), LV mass index (g/m^2^), tricuspid annular plane systolic excursion (TAPSE) (mm), systolic pulmonary artery pressure (PAPs) (mmHg), left atrial diameter (mm), E wave velocity (cm/sec), E/A ratio, deceleration time (DT) (mm), mitral annulus dimension (mm), inter-papillary distance (IPD) (mm) at the end of diastole and systole, diastolic and systolic sphericity index (SI) (calculated as the short- to long-axis ratio in apical 4ch-view) and systolic and diastolic conicity index (CI) (calculated as the ratio between the apical and the short axis to assess the shape of the apex) [9]. All measurements were done in triplicate and displayed as an average value. 

### 2.4. Follow-Up

The outcome of the study was considered all-cause mortality, including death within 30 days, during the follow-up period. Follow-up was 100% complete and was conducted either at the hospital during a routine clinical evaluation or by telephone contact with the patients, their relatives or their family doctors. If a patient was not seen in the hospital or a telephone interview was not possible, the National Registry of Death was contacted.

### 2.5. Surgical Technique

The surgical technique has been previously reported in detail [8]. Briefly, the procedure was performed under total cardiac arrest with antegrade crystalloid cardioplegia. After completion of coronary grafting when indicated, the left ventricle was opened with an incision parallel to the left anterior descending artery, starting at the middle scarred region and ending at the apex. Surgical ventricular reconstruction was performed using a mannequin (TRISVR, Chase Medical Richardson, TX, USA) filled at 50–60 mL/m^2^ to optimize the size and shape of the new ventricle. When needed, the mitral valve was repaired through the ventricular opening.

### 2.6. Statistical Analysis

Categorical variables were presented as number (%) and continuous variables as median [interquartile range]. Changes over time between preoperative and postoperative echocardiographic characteristics were compared with the McNemar paired test. 

The differences in baseline demographic and clinical characteristics between Group I and Group II were compared with the Chi-square test; continuous variables were compared by the non-parametric Kruskal–Wallis test for non-normally distributed data.

The association between demographic, preoperative echocardiographic variables and the time to all-cause death was investigated by the use of a univariate and multivariable Cox proportional hazard regression analysis. 

A first screening of potential predictors was performed by a univariate analysis. For this method, the model considered baseline demographic and preoperative echocardiographic characteristics significantly associated with the outcome and defined by a backward selection. The internal validity of the final models was partially assessed by the bootstrap resampling technique [10]. For each of the 200 bootstrap samples, the model was refitted and tested on the original sample to obtain estimates of predictive accuracy. The bootstrap resampling did not include the first stage of variable selection. 

The proportionality and linearity of hazards were evaluated by graphic inspection and testing for Martingale residuals. Median follow-up time was calculated according to the reverse Kaplan–Meier method.

All *p* values were two-tailed and considered significant if <0.05. Statistical analyses were done with SAS software, version 9.4 (SAS Institute, Inc., Cary, NC, USA) and with the R program (http://CRAN.R-project.org (accessed on 28 March 2022)) with the RMS package.

## 3. Results

The demographics and clinical profile data of the patient population are shown in Table 1, based on the two different periods. 

At baseline, although patients in Group I had less hypertension (203 [54.7%] versus 178 [64.2%], *p* = 0.0146), hyperlipidemia (188 [50.6%] versus 193 [69.7%], *p* < 0.0001) and tabagic habit (222 [59.8%] versus 217 [78.3%], *p* < 0.0001), they were more symptomatic for angina (176 [47.4%] versus 54 [19.5%], *p* < 0.0001), but less symptomatic for HF (NYHA class III/IV, 172 [46.3%] versus 158 [57%], *p* = 0.0071). 

Medical treatment at the time of admission is reported in Table 1. The prescription of ACE-inhibitors was similar between Group I and Group II (83.20% versus 81.82%, *p* = 0.6467), while β–blockers (64.23% versus 89.09%, *p* < 0.0001), aspirin (72.63 % versus 93.45%, *p* < 0.0001), diuretics (75.88 % versus 93.12%, *p* < 0.0001), statins (47.70% versus 93.48%, *p* < 0.0001) and oral anticoagulants (6.79% versus 11.39, *p* < 0.0001) were prescribed significantly more frequently in Group II. Conversely, Group I received more nitrates (42.82% versus 17.09%, *p* < 0.0001). PCI prior to surgery was performed less frequently in Group I (79 [21.2%] versus 104 [37.6%], *p* < 0.0001), while the arrhythmogenic burden was greater in Group II compared with Group I, with more ventricular arrhythmias (75 [27.1%] versus 31 [8.3%], *p* < 0.0001) and implanted ICD (25 [9%] versus 15 [4.0%], *p* < 0.0001), (Table 1).

At baseline echocardiography (Table 2), the EDV index (106 [88.8–128.1] mL/m^2^ versus 118.3 [88.7–140.8] mL/m^2^, *p* < 0.0001) and the ESV index (70.5 [55.7–92.0] mL/m^2^ versus 81.5 [65.8–101.9] mL/m^2^, *p* < 0.0001) were lower in Group I, but not the EF (33 [26–38] % versus 31 [25–36] %, *p* = 0.010). Group I patients had a higher LV mass (168.26 [141.9–203.8] g/m^2^ versus 160.5 [131.2–193.7] g/m^2^, *p* = 0.0060), a lower left atrial dimension (46 [40.5–50] mm versus 47 [43–52] mm, *p* = 0.0083), a lower E/A ratio (0.82 [0.64–1.40] versus 1.15 [0.72–2.09], *p* = 0.0003) and more anterior remodeling (316 [85.2%] versus 210 [75.8%], *p* = 0.0100), while the rate of posterior remodeling was higher (44 [18.7%] versus 52 [11.8%], *p* = 0.0100) in Group II. Grade 3/4 of mitral regurgitation was higher in Group II (40.4% versus 28.2%, *p* < 0.0001), which showed a higher SI.

The presence of 3-vessel CAD was higher in Group I (272 [73.3%] versus 164 [59.2%], *p* < 0.0001), resulting in more coronary grafts combined with SVR (347 [93.53%] versus 235 [84.84%], *p* = 0.0003) as well as a higher percentage of two or more distal anastomosis (278 [74.9%] versus 160 [57.7%], *p* < 0.0001) (Table 2).

**Figure 1:**
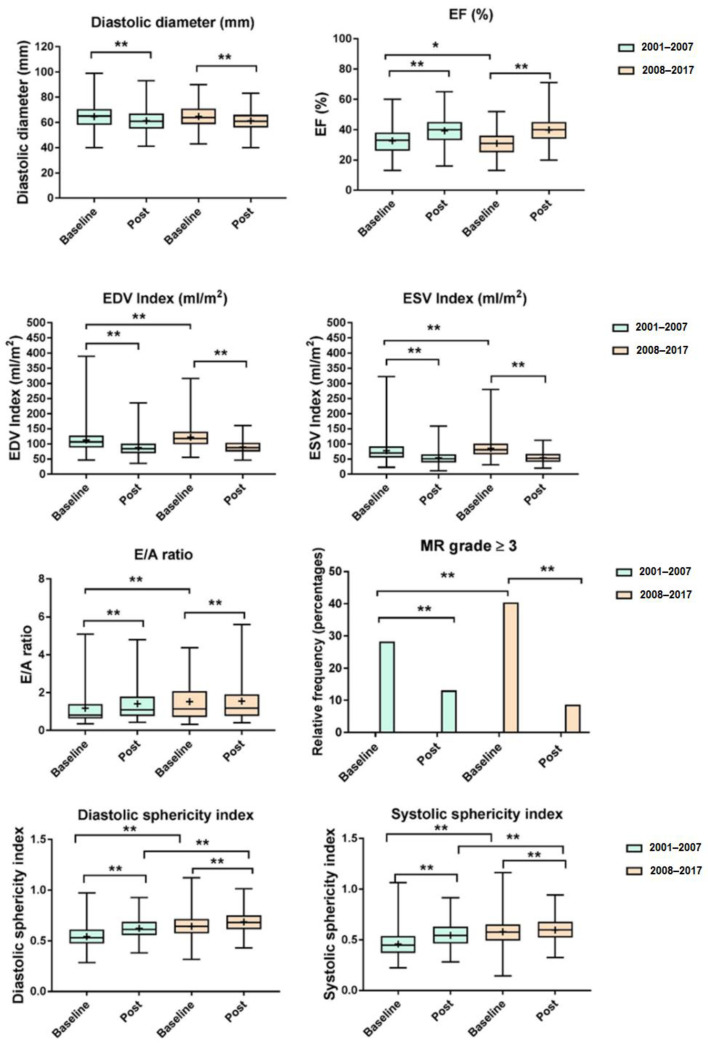
Boxplot for baseline and postoperative echocardiographic variables by period. *, *p* < 0.05; **, *p* < 0.001.

Figure 1 shows the changes after surgery in echocardiographic parameters in Group I and Group II in respect to baseline differences.

Postoperative variables by period: diastolic diameter (mm) *n* total = 454, *n* = 230 in [2001–2007] and *n* = 224 in [2008–2017]—EF (%) *n* total = 478, *n* = 239 in [2001–2007] and *n* = 239 in [2008–2017]—EDV index (mL/m^2^) *n* total = 466, *n* = 227 in [2001–2007] and *n* = 239 in [2008–2017]—ESV index (mL/m^2^) *n* total = 466, *n* = 227 in [2001–2007] and *n* = 239 in [2008–2017]—E/A ratio *n* total = 359, *n* = 173 in [2001–2007] and *n* = 186 in [2008–2017]—MR grade *n* total = 495, *n* = 252 in [2001–2007] and *n* = 243 in [2008–2017]—sphericity index, diastole *n* total = 397, *n* = 175 in [2001–2007] and *n* = 222 in [2008–2017]—sphericity index, systole *n* total = 396, *n* = 174 in [2001–2007] and *n* = 222 in [2008–2017].

Overall, operative mortality defined as mortality within 30 days from surgery was 6.6%, corresponding to 43 patients. Of these, 27 (7.3%) were in Group 1 and 16 (5.7%) in Group 2 (*p* = 0.4475). 

The mean follow-up time was 10 years (range, 0 days to 16 years) for the entire cohort; 13 years (range, 0 days to 16 years) in Group I and 5 years (range, 0 days to 10 years) in Group II. 

**Figure 2:**
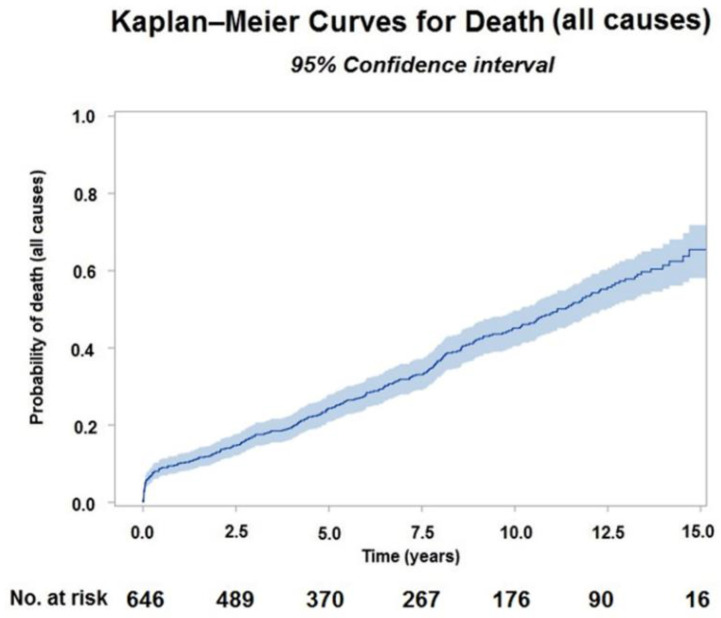
Kaplan-Meier curve for all-cause mortality for the total study cohort.

The Kaplan–Meier estimate for all-cause mortality of the entire population was 13% (10.5–15.8%) at 2 years, 19.2% (16.2–22.5%) at 4 years and 36.6% (32.4–40.9%) at 8 years (Figure 2).

**Figure 3:**
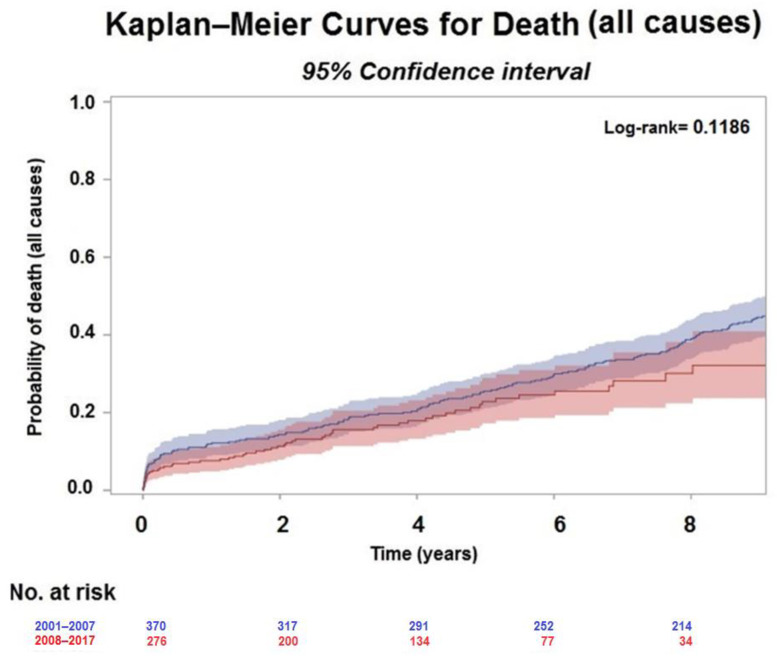
Kaplan-Meier curves for all-cause mortality by period.

The Kaplan–Meier estimate for mortality was 14.6% (11.2–18.4%) and 11.8% (8.2–16%) at 2 years, 20.6% (16.6–24.9%) and 18.5% (13.8–23.9%) at 4 years and 39% (34–44%) and 32.2% (23.7–40.9%) at 8 years (Figure 3), in Group I and Group II, respectively. There was no evidence of a difference in all-cause death between the two groups (Log-rank = 0.11).

In the multivariable Cox regression analysis, age (HR 1.069, 95% CI: 1.046–1.093, *p* < 0.0001), baseline EF (HR 0.97, 95% CI: 0.95–0.99, *p* = 0.0151) and E/A ratio (HR 1.61, 95% CI: 1.31–1.99, *p* < 0.0001) were significantly associated with mortality in Group I. Conversely, baseline NYHA class (HR 4.282, 95% CI: 1.474–12.441, *p* = 0.0075) and diastolic sphericity index (HR 1.03, 95% CI: 1.00–1.07, *p* = 0.0379) were found to be independent predictors of mortality in Group II (Table 3).

## 4. Discussion

This study reports the largest single-center experience on SVR over 16 years of observation. The major findings of this study include: (1) SVR, mostly combined with CABG in patients with ischemic systolic LV dysfunction, was carried out with a risk of death acceptably low at long-term follow-up; (2) in a real world ischemic HF population, the volume reduction was feasible even in patients with more extensive CAD (Group I); (3) in patients with worse LV remodeling and less extensive CAD (Group II), SVR showed a trend toward a better outcome, though not statistically significant. 

Although the comparison between populations of different studies is always hazardous because of differences in baseline characteristics or study design, our results portend an excellent prognosis for patients with ischemic HF and systolic LV dysfunction in terms of all-cause mortality, with a 4-year risk of death of 19.2% in the overall population, lower than other rates reported in the literature, including the STICH trial [5,7]. 

The surgical treatment of patients with ischemic HF has been a challenge for many years because of the paucity of data from randomized clinical trials mainly regarding patients with angina and significant CAD. The landmark STICH trial was designed to determine the benefit of CABG plus SVR in patients with ischemic LV dysfunction (Hypothesis 2 [7]). The primary outcome analysis failed to show a superiority of the combined procedure compared with CABG alone, calling into question the role of SVR in this high-risk population. Indeed, the STICH trial enrolled only a small percentage (about 20%) of the eligible population, limiting the generalizability of the results, especially in the absence of a concomitant registry. Furthermore, few non-pre-specified retrospective analyses of the STICH trial –Hypothesis 2 have been conducted due to the poor confidence of many surgeons with this procedure [11].

The results of our study share some insights with the STICH trial and, at the same time, focus attention on the selection of patients that could best benefit from this procedure.

Firstly, the Group I population had very similar results to the STICH population (enrollment period 2002–2005). As most patients were more symptomatic for angina and less for HF, they received less PCI before surgery and had more CAD that required a higher number of coronary grafts combined with SVR (Figure 3), resulting in a favorable post-surgical volume (median Δ ESVI equal to 28.7%). Although we cannot discern the weight of the individual procedures on the percentage of volume reduction, it is noteworthy that in this population the decrease in LV size was more important than that reported in the STICH trial [7] (ranging between 6% in the CABG group and 19% in the CABG plus SVR group), supporting a major role of SVR in inducing LV reverse remodeling compared to medical therapy and/or devices [12]. On the other side, despite the evident benefit in all-cause death at 2, 4 and 8 years, the complex ischemic burden related to the presence of extensive CAD and angina in this group of patients might have been the driver for the negative impact of the diastolic dysfunction on the outcome. This implies that an increased E/A ratio along with a low EF may confer a higher risk of death at follow-up, in agreement with previous observations [13,14].

**Figure 4:**
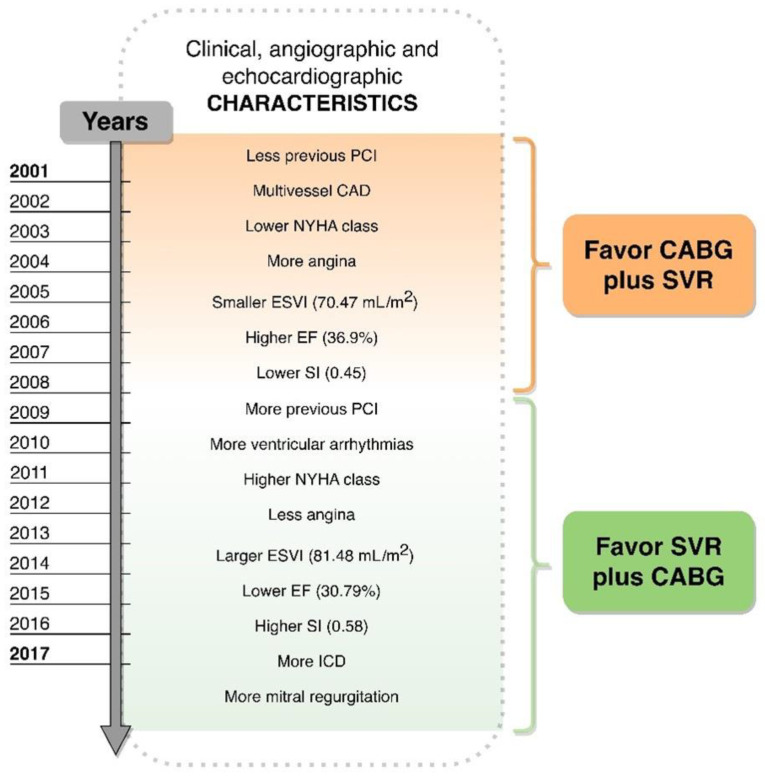
Temporal changes in clinical, angiographic and echocardiographic characteristics which may have influenced the decision for favoring SVR plus more or less coronary grafts.

Conversely, the Group II population presented more symptoms of HF, a higher arrhythmogenic burden likely driven by the LV dilatation and a less ischemic profile, apart from the aetiology, with a more extensive LV remodeling and more secondary mitral regurgitation causing a less favorable geometry (Figure 3 and Figure 4). 

In this group, a higher baseline LV diastolic SI, with the ventricle being even more spherical after surgery, was associated with worse survival (Figure 1). This observation is in agreement with the results from the STICH trial reported by Oh et al. [15]. However, the link between baseline SI, changes after surgery and survival is not completely clear because the STICH investigators ascribed the worsening of diastolic dysfunction and/or mitral regurgitation to an increase in SI. Conversely, our results show that, although SI increases after surgery, there is a significant improvement in mitral regurgitation (Figure 1). Moreover, changes in E/A ratio do not necessarily indicate a deterioration in diastolic function, meaning that the degree of dysfunction can be the same (grade II) beyond the statistical significance (Figure 1) [16]. Moreover, although the survival was not statistically different between groups, a trend toward a better outcome was observed in Group II, supporting a more robust role of SVR in patients with greater remodeling. Therefore, to better understand the link between changes in LV remodeling phenotypes, surgical technique and outcomes, a new analysis is needed.

This study has several limitations. First, the observation time was extremely long and included changes in patients’ profile, medical and percutaneous therapies; surgical techniques; and length of follow-up. The division used in this study, partially arbitrary, was an attempt to take into account, in some way, how the population has changed over the years. Although our center has a large amount of experience in this field, data collection suffered from missing information, including the total number of previous PCI, which could have affected changes in LV remodeling as well as the presentation of the patients. On the other side, the released STICH results in March 2009 have undoubtedly affected the decision to refer a patient for SVR, but the same results pushed expert surgeons to reconsider the indications [17]. 

However, despite these potential confounders, the overall number of patients and the length of the follow-up make the survival analysis fairly consistent. 

Lastly, our findings should be applied to other populations with ischemic HF with caution because of many differences in baseline characteristics, data collection and surgical expertise.

## 5. Conclusions

The patient population has consistently changed over time, making a shift from patients mainly symptomatic for angina and suitable for CABG to patients with prevalent symptoms of HF and worse LV remodeling. In the latter group, the therapeutic target might be the ventricle rather than coronary arteries. Suggested indications for SVR include: a previous anterior or posterior MI; a preoperative LV ESV index > 60 mL/m^2^; regional LV asynergy, either dyskinetic or akinetic; predominant HF symptoms [NYHA functional class III/IV] or in the presence of ventricular arrhythmias and/or angina needing surgical revascularization if the previous conditions are present. The procedure is controindicated in the presence of severe dysfunction of the right ventricle and/or severe diastolic dysfunction [8]. These criteria should guide the patient selection, making SVR still a possible therapeutic strategy for ischemic HF, along with the operator experience and the volume of treated patients [18]. Of course, further studies are required in order to establish the real benefits of SVR at the time of CABG.

## Figures and Tables

**Table 1:** Baseline characteristics of the total study cohort and by period.

	*n*	Total	*n*	2001–2007 (*n* = 371)	*n*	2008–2017	*p*-Value
Age, years	648	66 [58–72]	371	67 [58–72]	277	64 [58–71]	0.1365
BSA	648	1.83 [1.74–1.92]	371	1.83 [1.73–1.94]	277	1.85 [1.75–1.94]	0.0689
Creatinine	648	1.10 [0.91–1.39]	371	1.14 [0.96–1.44]	277	1.05 [0.88–1.30]	0.0020
Family history of CAD	648	271 (41.82)		132 (35.58)		139 (50.18)	0.0002
Smokers or ex-smokers	648	439 (67.75)		222 (59.84)		217 (78.34)	<0.0001
Hypertension	648	381 (58.80)		203 (54.72)		178 (64.26)	0.0146
Atrial fibrillation	648	91 (14.04)		49 (13.21)		42 (15.16)	0.4786
Stroke	648	55 (8.49)		42 (11.32)		13 (4.69)	0.0027
Angina	648	230 (35.49)		176 (47.44)		54 (19.49)	<0.0001
Ventricular arrhythmias	648	106 (16.36)		31 (8.36)		75 (27.08)	<0.0001
Chronic renal failure	648	47 (7.25)		24 (6.47)		23 (8.30)	0.3731
Diabetes mellitus	648	166 (25.66)		97 (26.15)		69 (25.00)	0.7414
Hypercholesterolemia	648	381 (58.80)		188 (50.67)		193 (69.68)	<0.0001
NYHA class III/IV	648	330 (50.93)		172 (46.36)		158 (57.04)	0.0071
Previous PCI		183 (28.24)		79 (21.29)		104 (37.55)	<0.0001
PCI + ICD		37 (5.21)		3 (0.81)		34 (12.27)	
ICD		40 (6.17)		15 (4.04)		25 (9.03)	
ACE inhibitor	644	532 (82.61)		304 (83.20)		225 (81.82)	0.6477
β-blockers	644	482 (74.84)		237 (64.23)		245 (89.09)	<0.0001
Aspirin	644	525 (81.52)		268 (72.63)		257 (93.45)	<0.0001
Digoxin	644	46 (7.14)		37 (10.03)		9 (3.27)	0.0010
Statins	645	434 (67.29)		176 (47.70)		258 (93.48)	<0.0001
Diuretic	645	537 (83.26)		280 (75.88)		257 (93.12)	<0.0001
Oral anticoagulant	644	73 (11.34)		25 (6.79)		48 (11.39)	<0.0001
Amiodarone	644	146 (22.67)		85 (23.04)		61 (22.18)	0.7981
Nitrates	644	205 (31.83)		158 (42.82)		47 (17.09)	<0.0001

Data are median [Q1–Q3] or number (%); BSA = body surface area; CAD = coronary artery disease; PCI = percutaneous coronary intervention; NYHA = New York Heart Association.

**Table 2:** Baseline echocardiographic, angiographic and operative variables of the total study cohort and by period.

	*N*	Total	*n*	2001–2007 (*n* = 371)	*n*	2008–2017 (*n* = 277)	*p*-Value
Diastolic diameter (mm)	630	65 [58–71]	356	65 [58–70.50]	274	64 [59–71]	0.8698
Systolic diameter (mm)	625	51 [44–59]	355	51 [44–59]	270	51 [45–59]	0.6611
EDV index (mL/m^2^)	645	112.12 [92.34–134.30]	368	106.68 [88.77–128.06]	277	118.27 [98.86–140.83]	<0.0001
ESV index (mL/m^2^)	646	76.08 [59.17–95.81]	369	70.47 [55.65–92.02]	277	81.48 [65.79–101.85]	<0.0001
EF (%)	648	32 [26–37]	371	33 [26–38]	277	31 [25–36]	0.0100
SV index (mL/m^2^)	644	35.20 [29.38–41.69]	367	34.69 [24.14–40.61]	277	36.57 [29.63–42.55]	0.1186
TAPSE (mm)	609	20 [18–23]	335	20 [17–23]	274	21 [18–24]	0.0158
PAPs (mmHg)	556	38 [30.5–48]	282	38 [32–46]	274	38 [30–49]	0.4370
LVMI (g/m^2^)	561	164.29 [137.88–199.48]	336	168.26 [141.93–203.81]	225	160.51 [131.22–193.68]	0.0060
RWT	597	0.32 [0.27–0.38]	347	0.33 [0.28–0.40]	250	0.29 [0.25–0.36]	<0.0001
Left atrial diameter (mm)	594	46 [41–51]	340	46 [40.50–50]	254	47 [43–52]	0.0083
E/A ratio	458	0.96 [0.66–1.71]	220	0.82 [0.64–1.40]	238	1.15 [0.72–2.09]	0.0003
DT (mm)	435	185 [149–239]	197	190 [155–231]	238	182 [144–254]	0.9478
Mitral annulus (mm)	367	34 [30–37]	162	35 [31–39]	205	32 [29–37]	0.0017
IPD, diastole (mm)	254	3 [2.5–3.5]	124	2.60 [2.20–3.1]	132	3.20 [2.8–3.6]	<0.0001
IPD, systole (mm)	254	2.10 [1.7–2.6]	124	1.77 [1.4–2.6]	132	2.30 [2.00–2.7]	<0.0001
Sphericity index, diastole	395	0.60 [0.50–0.68]	152	0.60 [0.50–0.68]	243	0.64 [0.57–0.72]	<0.0001
Sphericity index, systole	394	0.53 [0.43–0.62]	152	0.45 [0.37–0.54]	243	0.58 [0.49–0.65]	<0.0001
Conicity index, diastole	385	0.88 [0.80–0.96]	142	0.81 [0.73–0.95]	243	0.89 [0.83–0.97]	<0.0001
Conicity index, systole	385	0.94 [0.83–1.09]	142	0.95 [0.80–1.11]	243	0.93 [0.84–1.08]	0.8379
MR grade							
0		80 (12.35)		74 (19.95)		6 (2.17)	
1		209 (32.25)		126 (33.96)		83 (29.96)	
2		142 (21.91)		66 (17.79)		76 (27.44)	<0.0001
3		119 (18.36)		58 (15.63)		61 (22.02)	
4		98 (15.12)		47 (12.67)		51 (18.41)	
Site of remodeling							
Posterior		96 (14.81)		44 (11.86)		52 (18.77)	
Anterior		526 (81.17)		316 (85.18)		210 (75.81)	0.0100
Anterior & posterior		26 (4.01)		11 (2.96)		15 (5.42)	
Coronary angiography							
Single vessel disease		159 (24.54)		85 (22.91)		74 (26.71)	
Multivessel disease		436 (67.28)		272 (73.32)		164 (59.21)	<0.0001
No residual stenosis		53 (8.18)		14 (3.77)		39 (14.08)	
Mitral valve surgery		200 (30.86)		96 (25.88)		104 (37.55)	0.0015
CABG + SVR		582 (89.81)		347 (93.53)		235 (84.84)	0.0003
SVR		66 (10.19)		24 (6.47)		42 (15.16)	
Number of distal anastomosis							
0		66 (10.19)		24 (6.47)		42 (15.16)	
1		144 (22.22)		69 (18.60)		75 (27.08)	<0.0001
>=2		438 (67.59)		278 (74.93)		160 (57.76)	

Data are median [Q1–Q3] or number (%). EDV = end-diastolic volume; ESV = end-systolic volume; EF= ejection fraction; SV = stroke volume; TAPSE = tricuspid annular plane systolic excursion; PAPs = systolic pulmonary artery pressure; LVMI = left ventricular mass index; RWT = relative wall thickness; DT = deceleration time; IPM = inter-papillary distance; MR = mitral regurgitation.

**Table 3:** Univariate and multivariable Cox models for all-cause mortality.

		2001–2007 (*n* = 371)				2008–2017 (*n* = 277)			
		Univariate		Multivariate		Univariate		Multivariate	
		Death (*n* = 212)		Death		Death (*n* = 57)		Death	
	Risk Category	Hazard Ratio (95% IC)	*p*-Value	Hazard Ratio (95% IC)	*p*-Value	Hazard Ratio (95% IC)	*p*-Value	Hazard Ratio (95% IC)	*p*-Value
Sex	M vs. F	0.83 [0.60–1.17]	0.2899			0.94 [0.44–1.99]	0.8688		
Site of remodeling									
Ant/post	Ant/post vs. Ant	1.82 [0.89–3.71]	0.0991			2.61 [1.17–5.85]	0.0194		
Post	Post vs. Ant	1.52 [1.03–2.24]	0.0359			1.56 [0.79–3.07]	0.1996		
Previous MI	Yes vs. No	1.10 [0.76–1.59]	0.5986			1.48 [0.73–3.03]	0.2761		
Hypertension	Yes vs. No	1.04 [0.79–1.36]	0.7783			1.01 [0.60–1.73]	0.9739		
Dyslipidemia	Yes vs. No	0.66 [0.50–0.86]	0.0024			1.06 [0.60–1.87]	0.8449		
Diabetes	Yes vs. No	1.24 [0.92–1.66]	0.1578			1.96 [1.15–3.34]	0.0136	1.96 [0.94–4.08]	0.0728
Smoke	Yes vs. No	1.18 [0.90–1.56]	0.2340			0.92 [0.50–1.71]	0.7928		
Previous procedures									
	PCI vs. No	0.87 [0.61–1.24]	0.4436			0.57 [0.30–1.09]	0.0908		
	PCI + ICD vs. No	2.27 [0.56–9.20]	0.2493			1.33 [0.60–2.96]	0.4868		
	ICD vs. No	1.01 [0.49–2.05]	0.9849			1.79 [0.81–3.99]	0.1516		
	Other vs. No	1.48 [0.92–2.40]	0.1071			0.98 [0.14–7.22]	0.9831		
Ventricular arrhythmias	Yes vs. No	1.29 [0.81–2.04]	0.2842			0.758 [0.40–1.43]	0.3858		
Atrial fibrillation	Yes vs. No	1.47 [1.02–2.11]	0.0384			3.14 [1.79–5.49]	<0.0001		
Stroke	Yes vs. No	1.32 [0.88–1.99]	0.1864			1.63 [0.59–4.50]	0.3496		
Chronic renal failure	Yes vs. No	2.92 [1.87–4.56]	<0.0001			3.36 [1.74–6.50]	0.0003		
Angiography									
Single vessels	Single vessels vs.	0.67 [0.33–1.37]	0.2750	0.96 [0.35–2.59]	0.9317	0.91 [0.31–2.67]	0.8617		
	No significant stenosis								
Multivessel disease	Multivessels disease vs.	0.95 [0.48–1.86]	0.8761	1.63 [0.62–4.28]	0.3219	1.68 [0.66–4.27]	0.2728		
	No significant stenosis								
Angina	Yes vs. No	1.20 [0.91–1.57]	0.1885			0.83 [0.44–1.58]	0.5709		
NYHA	III-IV vs. I-II	1.82 [1.38–2.39]	<0.0001			3.12 [1.65–5.90]	0.0005	4.28 [1.47–12.44]	0.0075
MR grade	>= 2 vs. <2	1.41 [1.08–1.85]	0.0126			2.48 [1.25–4.92]	0.0092		
Age	1 unit	1.06 [1.04–1.07]	<0.0001	1.07 [1.05–1.09]	<0.0001	1.07 [1.03–1.10]	<0.0001	1.04 [0.99–1.09]	0.0815
BSA	1 unit	0.96 [0.43–2.14]	0.9241			0.07 [0.01–0.54]	0.0099		
Haemoglobin	1 unit	0.86 [0.78–0.94]	0.0005			0.83 [0.70–0.97]	0.0205		
Creatinine	1 unit	2.15 [1.78–2.59]	<0.0001			1.62 [1.21–2.18]	0.0012		
Diastolic diameter (mm)	1 unit	1.02 [1.00–1.04]	0.0126			1.03 [1.00–1.06]	0.0511		
Systolic diameter (mm)	1 unit	1.02 [1.00–1.03]	0.0122			1.03 [1.00–1.06]	0.0173		
EDV index (mL/m^2^)	1 unit	1.00 [1.00–1.01]	0.0906			1.00 [1.00–1.01]	0.4235		
ESV index (mL/m^2^)	1 unit	1.00 [1.00–1.01]	0.0070			1.00 [1.00–1.01]	0.1935		
EF	1 unit	0.96 [0.94–0.98]	<0.0001	0.97 [0.95–0.99]	0.0151	0.95 [0.92–0.96]	0.0051		
SV index (mL/m^2^)	1 unit	0.98 [0.97–0.99]	0.0421			0.98 [0.95–1.01]	0.2228		
RWT	1 unit	0.11 [0.03–0.53]	0.0054			0.09 [0.01–1.29]	0.0765		
LVMI (g/m^2^)	1 unit	1.00 [1.00–1.00]	0.2207			1.00 [1.00–1.01]	0.1060		
Left atrial diameter (mm)	1 unit	1.04 [1.02–1.06]	<0.0001			1.05 [1.01–1.09]	0.0146		
E/A ratio	1 unit	1.50 [1.23–1.84]	<0.0001	1.61 [1.31–1.99]	<0.0001	1.39 [1.09–1.77]	0.0079		
DT (m sec)	1 unit	0.99 [0.99–1.00]	0.0604			1.00 [0.99–1.00]	0.2290		
TAPSE	1 unit	0.94 [0.91–0.98]	0.0027			0.91 [0.84–0.97]	0.0053		
PAPs (mmHg)	1 unit	1.02 [1.01–1.03]	0.0006			1.02 [1.01–1.04]	0.0082		
Mitral annulus (mm)	1 unit	1.05 [1.02–1.09]	0.0051			1.05 [0.99–1.11]	0.0800		
IPD, diastole (mm)	1 unit	1.36 [1.09–1.68]	0.0058			1.34 [0.65–2.77]	0.4312		
IPD, systole (mm)	1 unit	1.47 [1.18–1.84]	0.0007			1.68 [0.89–3.17]	0.1061		
MR	Yes vs. No	1.73 [1.30–2.30]	0.0002			2.64 [1.56–4.47]	0.0003		
Diastolic sphericity index	1 unit	1.01 [0.99–1.03]	0.4265			1.04 [1.01–1.07]	0.0104	1.03 [1.00–1.07]	0.0379
Systolic sphericity index	1 unit	1.02 [1.00–1.03]	0.0931			1.04 [1.01–1.07]	0.0041		
Diastolic conicity index	1 unit	0.99 [0.98–1.00]	0.1062			0.98 [0.96–1.01]	0.1311		
Systolic conicity index	1 unit	0.99 [0.99–1.00]	0.1099			0.99 [0.97–1.00]	0.0552		

MI = myocardial infarction; PCI = percutaneous coronary intervention; NYHA = New York Heart Association; MR = mitral regurgitation; BSA = body surface area; EDV = end-diastolic volume; ESV = end-systolic volume; EF = ejection fraction; SV = stroke volume; RWT = relative wall thickness; LVMI = left ventricular mass index; DT = deceleration time; TAPSE = tricuspid annular plane systolic excursion; PAPs = systolic pulmonary artery pressure; IPM = inter-papillary distance.

## Data Availability

The data presented in this study are available on request from the corresponding author. The data are not publicly available due to the “Restricted Access” policy, as stated by Zenodo (the multi-disciplinary open repository maintained by CERN).
